# A Novel Detection Scheme for Motor Bearing Structure Defects in a High-Speed Train Using Stator Current

**DOI:** 10.3390/s24237675

**Published:** 2024-11-30

**Authors:** Qi Sun, Juan Zhu, Chunjun Chen

**Affiliations:** 1 Institute of Applied Electronics, China Academy of Engineering Physics, Mianyang 621900, China; 2 PLA Military Space Force, Mianyang 621900, China; 13650535983@163.com; 3 Technology and Equipment of Rail Transit Operation and Maintenance Key Laboratory of Sichuan Province, Chengdu 610031, China; kaunxin07@163.com

**Keywords:** high-speed train, traction motor bearing, fault diagnosis, stator current, deep belief network

## Abstract

Railway traction motor bearings (RTMB) are critical components in high-speed trains (HST) that are particularly susceptible to failure due to the high stress and rotational frequency they experience. To address the challenge of high false-positive rates in existing monitoring systems, this paper introduces a novel sensorless monitoring scheme that leverages stator current to detect fault-related characteristics, eliminating the need for additional sensors. This approach employs a hybrid signal preprocessing algorithm that integrates adaptive notch filtering (ANF) with envelope spectrum analysis (ESA) to effectively sparse the stator current and extract relevant fault features. A deep belief network (DBN) is utilized for the classification of the health status of the RTMB. To validate the scheme’s feasibility and effectiveness, we conducted experiments on a 1:1 scale high-speed railway traction motor, demonstrating that mechanical defects in RTMB can be reliably indicated by changes in stator current. Based on the analysis of experimental results, it was concluded that the fault detection accuracy of RTMB based on stator current is at least 17.3% higher than that of the fault diagnosis methods based on vibration in diagnosing whether the system has a fault. Among them, the method proposed in this paper is the best in diagnosing the presence and type of faults, with an accuracy that is at least 8.9% higher than other methods. This study not only presents a new method for RTMB monitoring but also contributes to the field by offering a more accurate and efficient alternative to current practices.

## 1. Introduction

**Figure 1:**
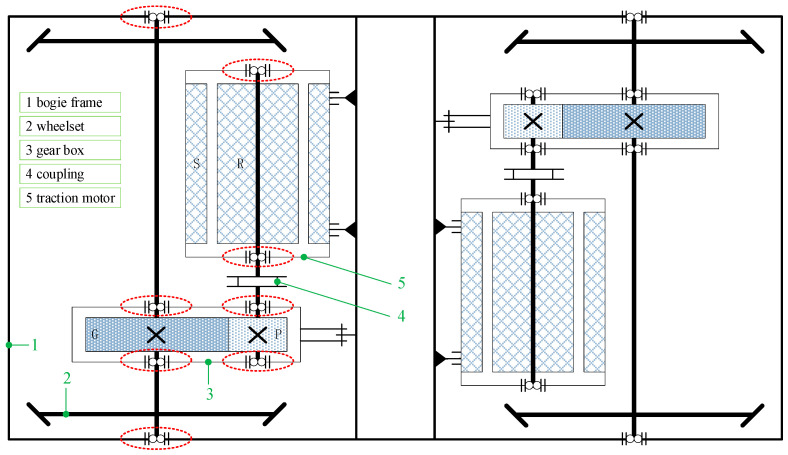
Plan view of railway bearings in bogie.

High-speed trains (HST) have become a popular mode of long-distance passenger transport worldwide due to their high speeds and environmentally friendly operation. Over the past three decades, China has developed approximately 200,000 km of rail network, including more than 39,000 km of high-speed rail, which accounts for over two-thirds of the world’s total. Today, Chinese railways have transitioned from an era of large-scale construction and manufacturing to a new era of smart operation and maintenance. Ensuring operational safety remains the top priority in the public service of high-speed trains. The rotating mechanical components in the bogie transmission system are particularly susceptible to structural failures due to the immense stress and high rotational speeds they endure during operation. The early detection of defects in railway rotating machinery is crucial for ensuring operational safety and preventing potential failures. These rotating components of the bogie include wheels [1], gears [2], and bearings [3]. One railway transmission device usually includes two wheelset bearings, four gear box bearings, and two traction motor bearings, as Figure 1 shows.

Over the past three decades, both academics and practitioners have extensively studied condition monitoring methods for rolling element bearings across various industries, leading to significant advancements and notable achievements in this field [4]. Based on the sensing technologies employed, these techniques can be divided into vibration-based [5], AE (acoustic emission)-based [6], sound-based [7], current-based [8], voltage-based [9], IAS (instantaneous angular speed)-based [10], and temperature-based [11] monitoring methods. In contrast to rolling element bearings used in general industrial applications, railway bearings operate in dynamic environments and are subject to additional sources of noise that can interfere with condition monitoring. As a result, not all bearing monitoring techniques are equally effective for railway bearings. For the railway field specifically [12], the temperature-based [13], AE-based [14], and vibration-based [15] methods have been drawn into bearing-fault diagnosis. The method of temperature monitoring is variable; however, its capability is limited to identifying faults in bearings that have progressed to a more advanced stage. Hence, it is always used as a supplement for the real-time monitoring of railway bearing nowadays. The AE monitoring technique usually has a high SNR (signal-to-noise ratio) for railway bearing monitoring [16], but it requires high sampling frequency (around 1 MHz), causing the pressure of data storage for the on-board detection application. As a result, the AE technique is frequently employed for wayside detection in contemporary railway monitoring systems [17]. The vibration monitoring approach is adept at identifying faults in their nascent stages and necessitates a sampling rate that is quite feasible (around 10 kHz). Hence, the vibration-based method is wildly used for the railway bearing structure health monitoring.

The vibration-based methods for railway bearing condition monitoring are mainly designed to enhance and extract fault characteristic frequency (FCF) components from motor housing vibration signals. For example, Cheng et al. [18] applied an improved minimum entropy deconvolution (MED) method using vibration signal for railway bearing-fault diagnosis. MED is used enhance the impulse-related component through an adaptive filter. The effectiveness of their proposed method was verified based on experimental data under inner-race, outer-race, and rolling element defect conditions and at the speeds at 200 km/h and 300 km/h, respectively. Yan Huang et al. [19] proposed a modified scale-space guiding variational mode decomposition (VMD) using vibration signals for high-speed railway bearing-fault diagnosis with an experimental speed of 100 km/h. The effectiveness of their proposed algorithm was proven by the data of the outer-race and roller defects. However, only single-point defects produce the four predictable FCFs, while generalized roughness produces unpredictable broadband effects on machine vibration [20]. Hence, the above-mentioned FCF-based detection method cannot cover all the failure situations for a real site application. Certain inherent characteristics of vibration signals impose inherent limitations on the use of vibration-based methods for monitoring the condition of railway bearings. Firstly, vibration signals are highly dependent on the placement of sensors. Secondly, these signals can experience significant attenuation as they travel through the transmission path and are susceptible to interference from wheel-rail impact noises, leading to numerous false alarms in practical use.

Motor current signature analysis (MCSA) is a well-developed technique for the mechanical structure health monitoring of induction machines in generic industrial fields [21]. Eren et al. [22] proposed a bearing damage detection method using motor stator current. They highlighted that defects in bearings generate specific vibrations, which in turn cause a modulation in the stator current. In the study, the effectiveness of their proposed method for detecting bearing defects on the outer race and cage were proven by a rig test. It is noteworthy that a cage fault is usually difficult to detect using vibration signals because the cage only comes into contact with the bearing rollers. Singh et al. [23] proposed a continuous wavelet transform (CWT)-based detection method of bearing faults in mechanical systems using motor stator current. They pointed out that the faulty bearings installed in induction machines do not directly alter airgap eccentricity, but they affect the resultant torque of an induction motor. The effectiveness was proven based on the condition of outer-race defects. In conclusion, motor current signature analysis (MCSA) is recognized as a non-intrusive diagnostic method that does not require the additional cost and complexity of installing extra transducers or specialized diagnostic equipment for bearings. Consequently, MCSA is often viewed as a superior option for monitoring bearings, particularly due to its cost effectiveness and the ease with which it can access hard-to-reach machinery in industrial settings [24].

The real-time monitoring of RTMB is crucial for the operation safety of HST [25]. Acknowledging the advanced state of motor current signature analysis (MCSA) for monitoring the mechanical integrity of induction motors and noting that all high-speed railway traction motors in China utilize this type of motor, as detailed in Table 1, this research introduces an innovative monitoring approach. By employing the analysis of stator current, the proposed scheme aims to offer an additional method to enhance the precision and economic viability of condition monitoring for railway traction motor bearings (RTMB). Because the early fault features of RTMB are too weak to be extracted directly and are always buried in motor supply frequency components, a hybrid algorithm based on ANF, ESA, and DBN is proposed to denoise, sparse, and classify the raw stator current data. To examine the effectiveness, an experiment with a 1:1 high-speed railway traction motor under different bearing conditions was carried out. Based on the analysis of the experimental results, it was concluded that the fault detection accuracy of RTMB using stator current is at least 17.3% higher than that of vibration-based fault diagnosis methods in determining whether a fault exists in the system. Among all the methods, the approach proposed in this paper achieved the best performance in diagnosing both the presence and type of faults, with an accuracy at least 8.9% higher than the other methods.

The contributions of this paper are summarized as follows: (1) This paper presents a novel sensorless monitoring approach for railway traction motor bearings (RTMB) that uses stator current to detect faults, negating the need for additional sensors. (2) In this paper, a hybrid fault diagnosis scheme, MCSA-ANF-ESA-DBN, is proposed for the condition monitoring of traction motor bearings in HST. This diagnosis scheme is a more accurate and efficient monitoring technique for RTMBs, enhancing current practices in the field.

## 2. Proposed Scheme

**Figure 2:**
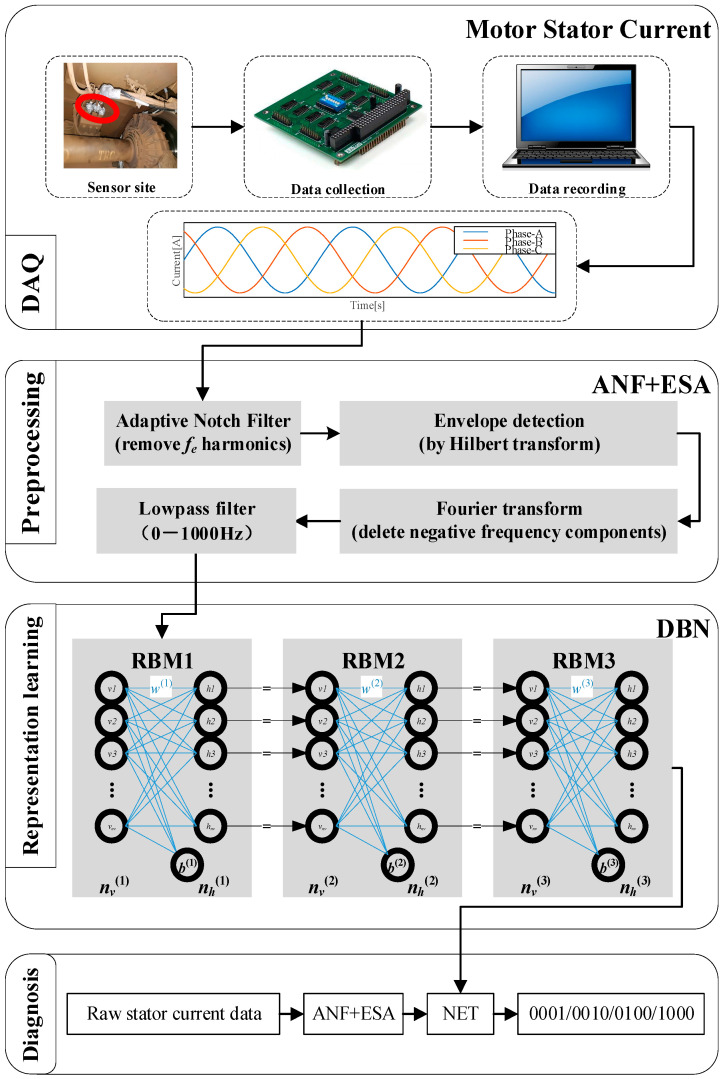
Proposed detection scheme.

In this paper, a hybrid fault diagnosis scheme, MCSA-ANF-ESA-DBN, is proposed for the condition monitoring of traction motor bearings in HST. The diagram of the proposed detection scheme is shown in Figure 2. In this section, the basic theories about MCSA, ANF, ESA, and DBN are described, and we further explain why these algorithms are used.

Through Figure 2, it can be seen first that motor stator currents were chosen to detect the RTMB faults. Then, the collected signals were filtered using ANF method to remove the strongest frequency component from stator current signals. Because the component at *f_e_* Hz is fault-unrelated and always existing in the stator current signal, a demodulation method was applied to the filtered signals to reveal the fault-related component in the frequency domain. At last, the processed signals were input into the DBN to classify health status.

### 2.1. Motor Current Signature Analysis (MCSA)

**Figure 3:**
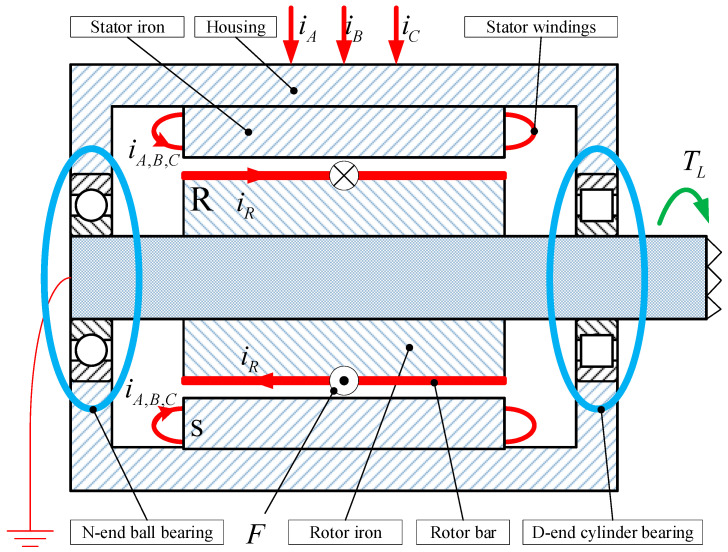
Section diagram of induction motor.

A diagram of axial section of the researched induction motor is shown in Figure 3, where the driving end is a cylindrical roller bearing, and the non-driving end is a ball roller bearing. Mechanical imbalance in induction motors can lead to the disturbance magnitude of the stator current [26].

According to D’Alembert’s principle, the mechanical balance on the motor rotor can be described as follows:

(1) $$\frac{J}{n_{p}}\frac{d\omega_{r}}{dt}=T_{e}-T_{L}-T_{f}$$

where *J* denotes the moment of inertia of the motor rotor, *n_p_* denotes the pole pairs of the motor, $\omega_{r}$ denotes the rotating speed of the motor shaft, *T_e_* denotes the electromagnetic torque on the rotor, *T_L_* denotes the load torque, and *T_f_* denotes the resistance moment of friction between the bearing and shaft. Upon the failure of a bearing, the frictional resistance torque between the bearing and the shaft, denoted as *T_f_*, increases, leading to a variation in the stator current [23]. However, the change in magnitude that the structural defect will have on the current is closely related to the mechanical parameters (including moment of inertia of rotor, frequency of the torque fluctuation, etc.) of the measured motor [27]. Hence, for a specific induction machine, further observation is needed to determine if the stator current-based method is effective for mechanical fault diagnosis.

### 2.2. Adaptive Notch Filter (ANF)

**Figure 4:**
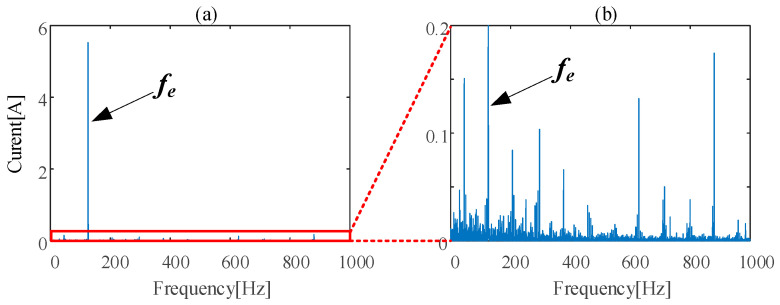
(**a**) Frequency characteristics of stator current of IM. (**b**) the local enlarged plot from (**a**).

Supply frequency, *f_e_*, is dominant in the frequency domain of stator current data, as Figure 4a shows. the local enlarged plot from Figure 4a is shown in Figure 4b. From Figure 4b, it is obvious that stator current contains abundant information. To eliminate the effect of the dominant fault-independent supply frequency component, an adaptive notch filter (ANF) was utilized to preprocess the raw stator current signal.

A notch filter is usually used as the tool of mode suppression in the control system of power electronics [28]. In this paper, a notch filter (NF) is drawn into mechanical defect diagnosis for RTMB. A notch filter is a second-order IIR filter. The transform function can be described as follows:

(2) $$H(z)=\frac{1-2\cos(\omega_{0})z^{-1}+1}{1-2R\cos(\omega_{0})z^{-1}+R^{2}}$$

**Figure 5:**
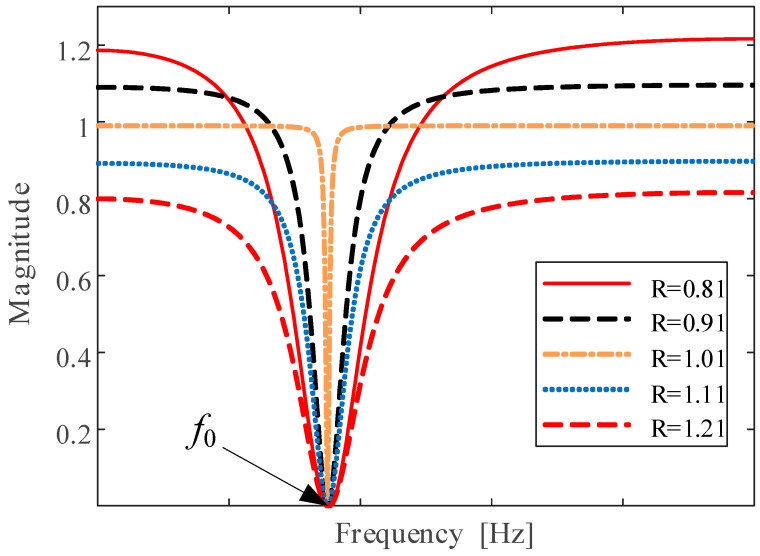
Amplitude characteristic of notch filters in the frequency domain.

Its differential form is as follow:

(3) $$y(n)=2R\cos\omega_{0}y(n-1)-R^{2}y(n-2)+2\cos\omega_{0}x(n-1)+x(n-2)-x(n)$$

where $\omega_{0}$ is the center frequency of the narrow stopband of NF, R denotes the shape parameter of NF, and the frequency characteristics of notch filter with different values of *R* are shown in Figure 5. Many wonderful studies have been completed on adaptive notch filters (for example, [29]). To deduce the computational cost, in this paper, the parameter R is fixed, and the parameter $\omega_{0}$ is adaptively matched with the varying train speed. More specifically, the center frequency of the narrow stopband of the notch filter is set at the following:

(4) $$\omega_{0}=\frac{2\pi }{f_{s}}\cdot f_{e}=\frac{2\pi }{f_{s}}\cdot \frac{n_{p}z_{g}f_{w}}{z_{p}}$$

where *f_s_*, *f_e_*, and *f_w_* denote the sampling frequency of the stator current signal, the power supply frequency of the motor, and the rotating frequency of wheel; *z_p_* and *z_g_* denote the tooth number of the pinion and gear, respectively.

### 2.3. Envelope Spectrum Analysis (ESA)

Mechanical faults of bearings can lead to amplitude modulations and/or frequency modulations (AM-FM) in vibration signals, according to both the theoretical model and experimental measurement [30]. Envelope analysis, also known as the demodulation technique, can extract a fault-related low-frequency signal from high-frequency harmonics for bearing-fault diagnosis. In this paper, envelope extraction is completed by Hilbert transform (HT). The HT of a signal *x*(*n*) is defined by the following:

(5) $$H[x(t)]=x(t)*\frac{1}{\pi t}=\int_{-\infty }^{\infty }x(\tau )\frac{1}{\pi (t-\tau )}d\tau =\frac{1}{\pi }\int_{-\infty }^{\infty }\frac{x(\tau )}{\left(t-\tau \right)}d\tau$$

where * represents the convolution operation. Based on HT, an analytic signal, z(t), can be formed by the following:

(6) $$z(t)=x(t)+jH[x(t)]=A(t)e^{j\phi (t)}$$

where the instantaneous amplitude, A(t), is calculated by the following:

(7) $$A(t)=\sqrt{x^{2}(t)+H{\left[x(t)\right]}^{2}}$$

Then, envelope spectrum (ES) is as follows:

(8) H(ω)=Re∑i=1nA(t)exp(j)∫ω(t)dt

### 2.4. Deep Belief Network (DBN)

The decision-making part of a traditional detection method for rolling element bearing-fault diagnosis is usually a fixed threshold [31]. However, the vibration signal of a traction motor has high nonlinearity, non-stationarity, and background [25]. A fixed threshold has limits in the robustness performance of structural health monitoring for RTMB. Hence, an intelligent method with DBN is proposed to address the issue. DBN is a dynamic Bayesian network, so it has a good fault-tolerance performance during the decision-making process.

**Figure 6:**
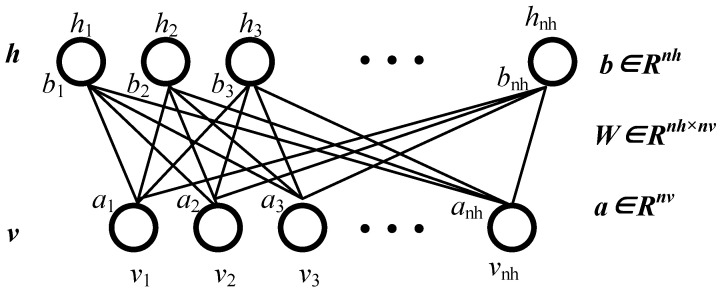
Architecture of RBM.

DBN is composed of multiple restricted Boltzmann machines (RBM) and a top-layer classifier [32]. As Figure 2 shows, three RBMs are used in this paper. Its architecture is shown in Figure 6, where ***v*** and ***h*** denote visible units and hidden units of the network; $v_{i}\in \{0,1\},h_{j}\in \{0,1\}$, and a, b denote the bias values of the network.

RBM assumes that the joint distribution of visible units and hidden units is in accordance with canonical distribution, which is described as follows:

(9) $$P(v,h)=\frac{1}{Z}e^{-E(v,h)}$$

where *Z* denotes the partition function that adjusts the probability sum to 1, and *E* denotes the network system energy. While assuming the network meets the features of the stochastic process that the Ising model describes, the energy of the network system can be expressed by the following:

(10) $$E(v,h)=-\sum_{i}a_{i}v_{i}-\sum_{j}b_{j}h_{j}-\sum_{i}\sum_{j}h_{j}w_{i,j}v_{i}$$

A basic common concept for a random system is that less energy means more stability. Hence, the RBM undergoes unsupervised training by minimizing the energy *E*. According to Equation (9), this is equal to maximizing the possibility *P*. The optimization objective of RBM is described as follows:

(11) $$L_{\theta ,S}=\prod_{n=1}^{n_{s}}P\left(v^{n}\right)$$

where θ={w,a,b} denotes weights and bias values of RBM network; $S=\left\{v^{1},v^{2},\cdots ,v^{n_{s}}\right\}$ denotes the input dataset; *n_s_* denotes the number of input sample sets. As for the process of updating weights and bias, the fast algorithm, i.e., contrastive divergence (CD), based on the concept of MCMC (Markov chain Monte Carlo) is used [33]. The top-layer classifier of DBN is a generic BP network in this paper. More specific information about DBN can be found in Meng et al. [34] and Salakhutdinov and Hinton [35].

## 3. Experiment and Datasets

### 3.1. Experimental Equipment

**Figure 7:**
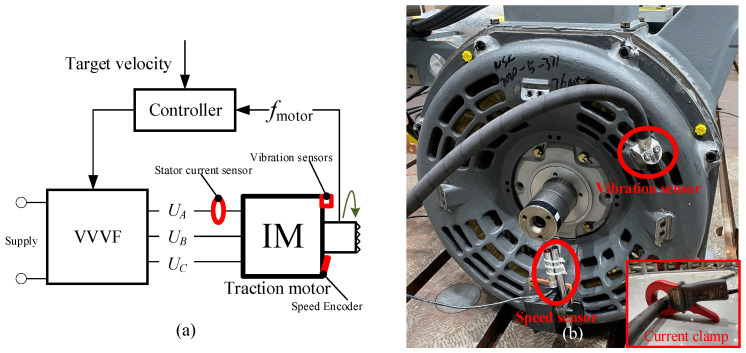
Diagram of the test bench and sensor locations. (**a**) The diagram of the test bench; (**b**) sensor location.

The experiment was implemented in a professional railway electrical motor test room, which is hemi-anechoic and can effectively protect the test motor from the exterior EMI (electromagnetic interference) effectively. The diagram of the test bench is shown in Figure 7a. The test motor was fed by an AC inverter controlled based on motor speed. The monitoring contents included the stator current, motor housing vibration acceleration, and motor angular speed. Their sensor location is shown in Figure 7b. Specially, the motor housing vibration was monitored for the comparative analysis in the next section. Its sampling frequency was 10 kHz. The speed and stator current signal are required for the proposed method. Their sampling frequencies were 100 kHz and 200 kHz, respectively. The test induction motor, a three-phase squirrel-cage induction motor, is the traction motor of a real HST. Its rated power is 625 kW, rated frequency 138 Hz, and rated current 155 A.

The primary task of signal acquisition is to collect the analog signals of the stator current in the traction motor. To achieve electrical isolation, a closed-loop current sensor based on the Hall effect was used. This component integrated a Hall element, a transformer, a magnetic amplifier, and electronic circuits, providing measurement, protection, and feedback functions. The sensor met the parameters found in Table 2.

Since the current sensor is a transformer-type current sensor and requires the collector to supply power to the sensor, the collector should meet the parameters in Table 3.

**Figure 8:**
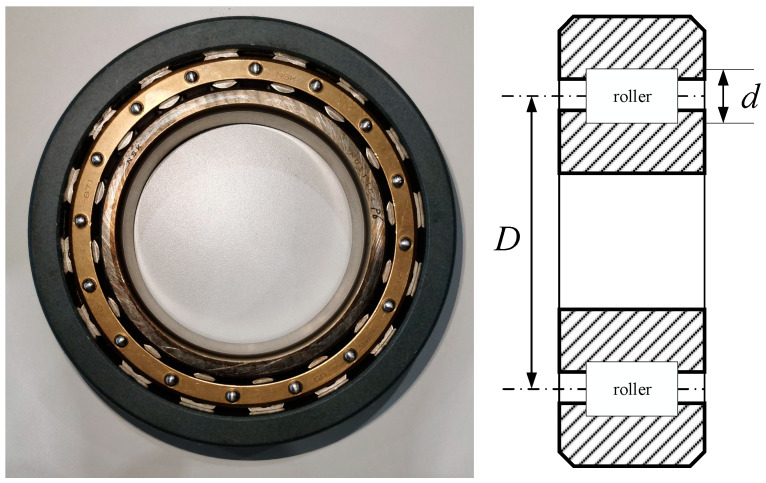
Physical image and cross-sectional schematic diagram of the researched bearing.

The studied bearing was the cylindrical roller bearing at the driving end of the traction motor in HST, shown as Figure 8. The number of rollers, *Z*, is 16. The roller diameter, *d*, is 13 mm. The roller pitch diameter, *D*, is 97.5 mm. The local pressure angle, α, is 0°.

### 3.2. Experimental Conditions

To observe the vibration response and stator current behavior of the railway traction motor under the excitation of a faulty bearing, the loading influence was ignored in this study. Usually, railway industry insiders define high-speed rails as systems of rolling stock and infrastructure that regularly operate at or above 250 km/h on new tracks or 200 km/h on existing tracks. The involved train in this paper usually runs on new tracks, and its maximum regular operation speed is 350 km/h. Its nominal wheel diameter is 0.88 m, its pinion tooth number is 29, and its gear tooth number is 73. The relationship between the motor rotating speed $\omega_{motor}$ and wheel forward speed $v_{wheel}$ is as follows:

(12) $$v_{wheel}=3.6\frac{z_{1}}{z_{2}}\frac{\omega_{motor}}{60}\pi D_{\mathrm{wheel}}$$

where D_wheel_ denotes the nominal diameter of wheel; *z*_1_ and *z*_2_ denote the tooth number of the pinion and gear. The units of $\omega_{motor}$ and $v_{wheel}$ are r/min and km/h. The wheel forward speed conditions were set at 250 km/h, 330 km/h, and 350 km/h. Their corresponding motor speeds were 3772 r/min, 4929 r/min, and 5278 r/min, respectively.

**Figure 9:**
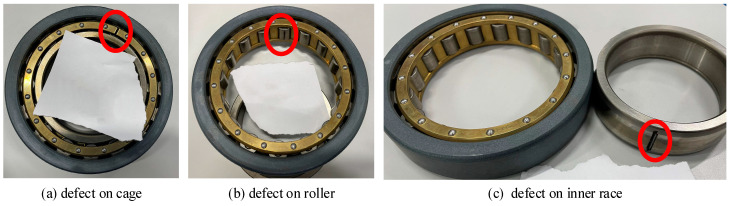
Bearing fault conditions.

The bearing is composed of four types of components: the cage, rollers, outer race, and inner race, respectively. Each of these defects is capable of producing a specific type of dynamic response. Three faulty conditions, as Figure 9 shows (the red circle in the picture is to indicate the location of the fault.), and a healthy condition were investigated in this paper. Creating an artificial defect on the outer race of a cylindrical bearing without disassembling it is highly challenging. Therefore, this paper does not address the issue of defects in the bearing’s outer race.

### 3.3. Data Sets

Four bearing health conditions were investigated in this study, and three different speed conditions were investigated under each bearing health condition. The total was 12 conditions, as shown in Table 4. To balance the radio of positive samples to negative samples, 100 samples were collected at each faulty condition and 200 samples at each healthy condition. The diagnosis time step was one second. Hence, the dimension of each raw sample is 200,000. After the ANF + ESA preprocessing and truncating processing, the dimension of input to the RBM1 is 1000. The input size is 1000 × 1500.

## 4. Study Results

### 4.1. Vibration-Based vs. Current-Based

**Figure 10:**
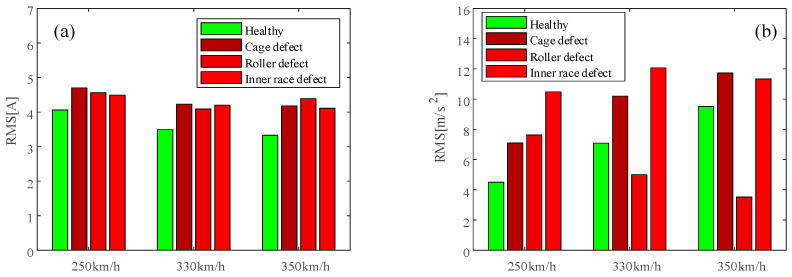
RMS of (**a**) raw stator current data; (**b**) raw motor housing vibration.

The time domain statistical characteristic, RMS (root mean square), of the raw motor stator current and raw motor housing vibration under 12 conditions is shown in Figure 10a,b. As shown in Figure 10a, the RMS values of the raw stator currents under faulty conditions are higher than in the healthy conditions no matter the speed level. As shown in Figure 10b, the motor housing vibration RMS values of the R-330 and R-350 conditions are even lower than the healthy conditions, which is unexpected, but the phenomenon that a rise in RMS value is not necessary in certain types of damage has already been pointed out by others [15]. From Figure 10, it can be observed that the RMS values calculated from the stator current are higher and more stable compared to the values obtained under non-faulty conditions. However, the RMS values obtained from vibration calculations are less stable, with some being higher and some lower than the RMS values obtained under non-faulty conditions.

**Figure 11:**
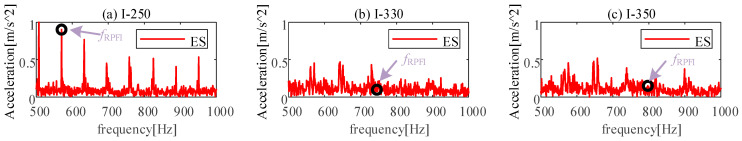
Diagnosis results based on ESA + FCF (TP rate is 1/3).

Most existent methods for railway bearing condition detection are designed to extract FCF harmonics in vibration signals. However, an inherent disadvantage of the method is that FCF may be overlapped by a strong resonance band, in which case it is hardly possible to extract the fault-related harmonic component by common demodulation algorithms. To explain the point, three inner-race defect cases were further analyzed. The FCF of an inner-race defect is calculated by the following:

(13) $$f_{\mathrm{RPFI}}=\frac{1}{2}Z\left(1+\frac{d}{D}\cos\alpha \right)\frac{\omega_{\mathrm{motor}}}{60}$$

where *f*_RPFI_ stands for the roller passing frequency of the inner race. With the geometry size of the test bearing substituted into Equation (13), the *f*_RPFI_ frequencies of I-250, I-330, and I-350 are 569.99 Hz, 744.83 Hz, and 797.56 Hz, respectively. The results of the captured FCF components after the demodulation preprocessing of the vibration signal are shown in Figure 11. Only the FCF component of I-250, shown in Figure 11a, could be captured. this means the detection accuracy rate for the inner defect is only 1/3 by the ESA + FCF vibration-based method.

**Figure 12:**
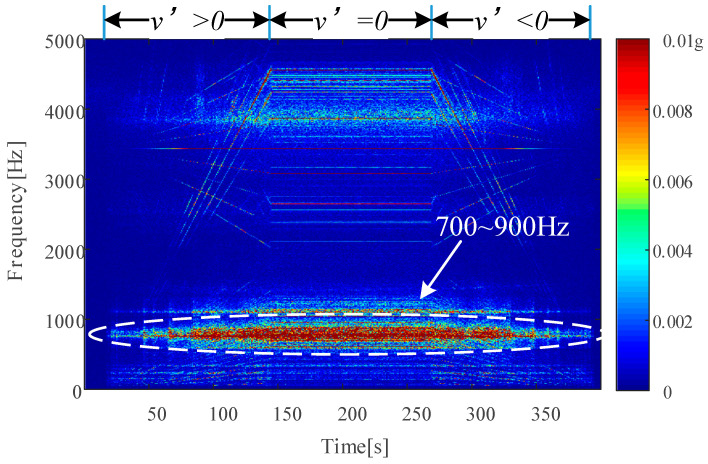
Time–frequency spectrum of vibration of H-350.

To further analyze the reason for this, a time–frequency (TF) analysis of the motor housing vibration signal during an acceleration–uniform–deceleration process under healthy bearing conditions at 350 km/h was completed by short-time Fourier transform, as shown in Figure 12, where ***v***’ denotes the vehicle acceleration. A strong resonance band at the range of 700 Hz to 900 Hz was found, which just overlaps with the FCF of I-330 and I-350, i.e., 744.83 Hz and 797.56 Hz. This is why the *f*_RPFI_ components disappeared in Figure 11b,c.

**Figure 13:**
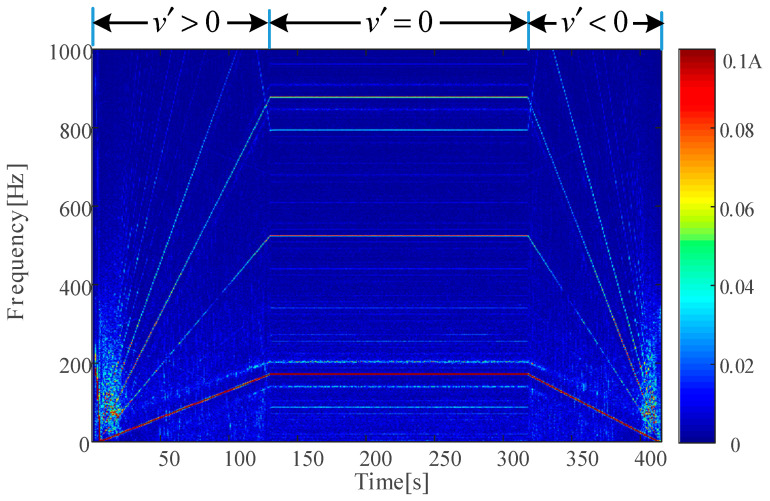
Time–frequency spectrum of current of H-350.

However, the spectrum of the stator current signal is usually sparse, with no strong resonance band within 1000 Hz. The time–frequency spectrum of the motor stator current during an acceleration–uniform–deceleration process under healthy conditions at 350 km/h is shown in Figure 13. Through a comparative analysis on the time–frequency spectrum of the vibration signal and current signal, we preliminarily proved that the stator current-based detection method can avoid the downside of the vibration-based method, which is easily mixed with the system features of the vibration transmission path.

### 4.2. Without ANF VS with ANF

**Figure 14:**
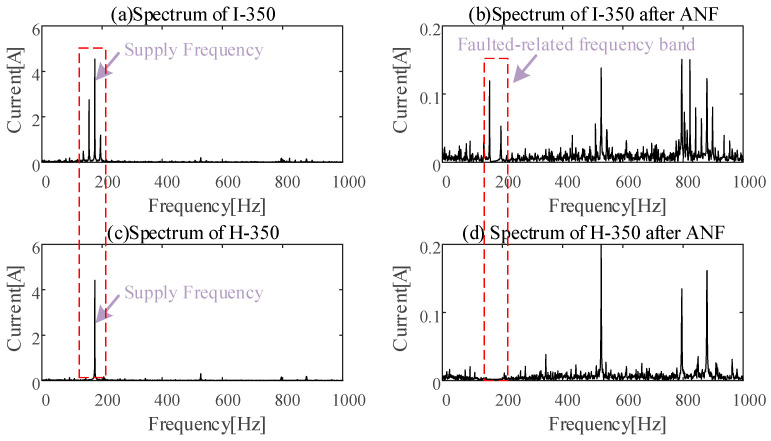
Healthy and unhealthy spectrum with/without ANF.

The dominant frequency of the stator current signal is the power supply frequency whether under healthy or unhealthy conditions. The stator current spectrum under a faulty condition and a healthy condition is shown in Figure 14a,c, respectively. Even though the bearing inner-race defect induces sidebands around the power supply frequency, the main frequency bands of the healthy data and faulty data are still similar. The corresponding spectrum after ANF preprocessing is shown in Figure 14b,d. Through the spectrum analysis of the stator current with and without ANF preprocessing, it was concluded that ANF is helpful in eliminating the influence of the power supply frequency component.

### 4.3. Without ESA vs. with ESA

**Figure 15:**
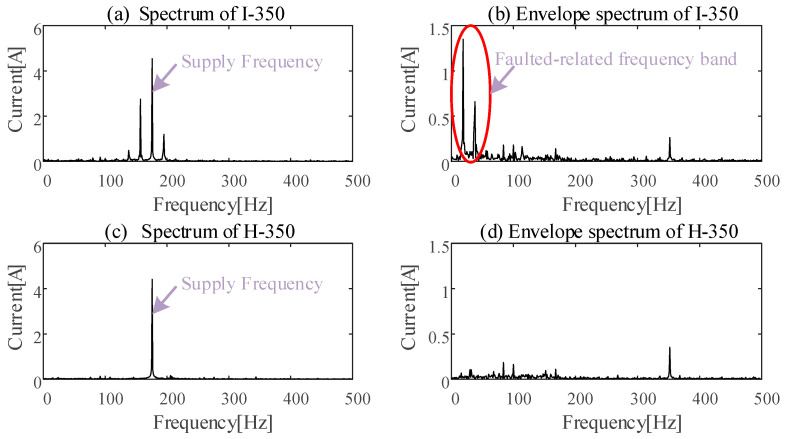
Spectrum vs. ES at healthy and unhealthy conditions.

According to dynamic models and the experimental measurements of bearings, the structure faults lead to amplitude and/or phase modulations of current signals [30]. The spectra under the inner-race defect and under healthy bearing conditions are shown in Figure 15a,c. Using the demodulation tool of HT to deal with the corresponding raw stator currents, the envelope spectra are shown in Figure 15b,d. According to the Figure 15, it can be concluded that ESA is helpful in separating the fault-related frequency bands from the noise bands.

### 4.4. Scheme Performance

To verify the effectiveness and superiority of the proposed scheme for the detection of high-speed railway motor bearing health conditions, it was compared with two existent vibration-based detection schemes: direct DBN-based and BPNN-based schemes. The accuracy rate is shown in Table 5.

The experimental results indicate that RTMB, based on stator current, achieves a fault detection accuracy that exceeds vibration-based methods by at least 17.3% when determining whether a fault exists. Additionally, the approach proposed in this paper is the most effective for diagnosing both the presence and type of faults, offering an accuracy improvement of at least 8.9% over other methods.

## 5. Conclusions

The primary objective of this research was to integrate the (MCSA)-based detection technique into the high-speed railway sector to enhance the accuracy of diagnosing the health status of traction motor bearings. The key contribution of this study is the development of an effective monitoring scheme for the health of traction motor bearings, the viability and efficacy of which were demonstrated through experimental research using a full-scale high-speed traction motor. The experimental results show that utilizing stator current outperforms the vibration-based methods by at least 17.3% in accurately detecting the presence of faults. Moreover, the method introduced in this paper provides the highest accuracy for both fault detection and fault type classification, surpassing other methods by a minimum of 8.9%.

The proposed detection scheme is effective and holds great promise in the field of RTMB fault detection. However, it is not without limitations. For example, DBN is computationally intensive, which takes a considerable amount of time to obtain an effective network model. To address this, we plan to optimize the classification algorithm within the detection scheme.

## Figures and Tables

**Table 1:** Application state of traction motor of Chinese HST.

Train	Release Year	Maximum Speed	Traction Motor	Manufacturer	City	Country
CRH_1_	2007	200 km/h	Three-Phase Asynchronous Squirrel cage Induction Motor	Bombardier Sifang (Qingdao) Transportation Ltd.	Qingdao	China
CRH_2_	2007	200 km/h→300 km/h		China South Locomotive&Rolling Stock Corporation Sifang Co., Ltd.	Qingdao	China
CRH_3_	2007	330 km/h		CSR Zhuzhou Electric Co., Ltd.	Zhuzhou	China
CRH_5_	2007	200 km/h→300 km/h		Changchun Railway Vehicles Co., Ltd.	Changchun	China
CRH380	2010	380 km/h		Zhuzhou CRRC Times Electric	Zhuzhou	China
CR400	2017	350 km/h		Zhuzhou CRRC Times Electric	Zhuzhou	China

**Table 2:** Sensor Performance Parameters.

Performance Specifications	Parameters
Power Supply Voltage	±15 V
Current Consumption	±15 mA
Insulation Resistance	>1000 MΩ
Accuracy	<±1%
Measurement Range	0~1000 A

**Table 3:** Collector Performance Parameters.

Performance Specifications	Parameters
Input Method	GND, SIN-DC, DIF-DC, AC, DIF-IEPE, SIN-IEPE
Voltage Range	±10 V, ±5 V, ±2 V, ±1 V, ±500 mV, ±200 mV, ±100 mV
Bridge Supply Voltage	2 VDC, 5 VDC, 10 VDC, 24 VDC
Bridge Configuration	Full Bridge, Half Bridge, Three-Wire 1/4 Bridge
Frequency Response Range	DC~100 kHz (+0.5 dB~−3 dB)

**Table 4:** Conditions of data samples.

Condition	Sample No.	Bearing	Speed	Label
1	0001–0200	Healthy	3772 r/min	H-250
2	0201–0400		4929 r/min	H-330
3	0401–0600		5278 r/min	H-350
4	0601–0700	Cage defect	3772 r/min	C-250
5	0701–0800		4929 r/min	C-330
6	0801–0900		5278 r/min	C-350
7	0901–1000	Roller defect	3772 r/min	R-250
8	1001–1100		4929 r/min	R-330
9	1101–1200		5278 r/min	R-350
10	1201–1300	Inner race defect	3772 r/min	I-250
11	1301–1400		4929 r/min	I-330
12	1401–1500		5278 r/min	I-350

**Table 5:** Accuracy of different detection schemes.

Data	Algorithms	Classification Accuracy [%]				
		Healthy	Cage Defect	Roller Defect	Inner Race Defect	Mean
Housing vibration	MED + ESA [18]	66.7	33.3	0	33.3	33.3
	VMD + ESA [19]	33.3	33.3	0	33.3	25.0
Stator current	DBN	84.0	19.2	13.0	4.3	38.2
	ANF + ESA + BPNN	100	84.2	94.9	75.5	91.1
	ANF + ESA + DBN	100	100	100	100	100

## Data Availability

The data supporting the findings of this study are not publicly available due to privacy.
